# Ecofriendly PEF- and PBF-Based Blends with Epoxidized Natural Rubber: Unraveling the Structure–Property Relationship

**DOI:** 10.3390/ma18174040

**Published:** 2025-08-28

**Authors:** Sandra Paszkiewicz, Konrad Walkowiak, Izabela Irska, Jakub Śmigielski, Elżbieta Piesowicz, Aleksander Hejna, Beata Dudziec, Mateusz Barczewski

**Affiliations:** 1 Department of Materials Technologies, Faculty of Mechanical Engineering and Mechatronics, West Pomeranian University of Technology, Piastow Av. 19, 70-310 Szczecin, Poland; wk42388@zut.edu.pl (K.W.); izabela.irska@zut.edu.pl (I.I.);; 2 Institute of Materials Technology, Faculty of Mechanical Engineering, Poznan University of Technology, Piotrowo 3, 61-138 Poznan, Poland; aleksander.hejna@put.poznan.pl (A.H.); mateusz.barczewski@put.poznan.pl (M.B.); 3 Department of Organometallic Chemistry, Faculty of Chemistry and Centre for Advanced Technologies, Adam Mickiewicz University, ul. Uniwersytetu Poznanskiego 10, 61-614 Poznan, Poland; beata.dudziec@gmail.com

**Keywords:** poly(ethylene 2,5-furandicarboxylate), biobased polyesters, epoxidized natural rubber, polymer blends, melt polycondensation, mechanical properties, miscibility

## Abstract

Two series of environmentally friendly polymer blends of bio-based poly(ethylene 2,5 furanoate) (PEF) and poly(butylene 2,5 furanoate) (PBF) with epoxidized natural rubber (epNR) have been prepared. Both bio-based polyesters were synthesized from dimethyl furan-2,5-dicarboxylate (DMFDC) and 1,2-ethylene glycol (EG) or 1,4-butylene glycol (BG) by a two-stage melt polycondensation process. The miscibility of the components in the blend was assessed using calculations based on Hoy’s method. The chemical interactions, presence of functional groups, miscibility, and possible reactions or cross-linking between polyesters and epNR were analyzed by Fourier Transform Infrared Spectroscopy (FTIR). A significant influence of epNR addition on the melt flow index (MFI), limited viscosity number (LVN), and apparent cross-link density values was also demonstrated. Phase transition temperatures and associated thermal phenomena in polyester/epNR blends were evaluated using differential scanning calorimetry (DSC) and thermogravimetric analysis (TGA). Oxidation onset temperature (OOT) tests were performed to obtain valuable information about the thermal-oxidative stability of the blends. Tensile tests revealed that the addition of epNR to PEF increases flexibility but at the same time reduces stiffness and tensile strength, especially at higher contents of epNR. In the case of PBF, a gradual decrease in tensile strength and elastic modulus is observed with increasing epNR content. Additionally, hardness tests showed that the addition of epNR leads to a decrease in hardness for both PEF- and PBF-based compositions.

## 1. Introduction

The development of commercial polymers can proceed through three primary synthetic strategies to meet market demands and expand potential applications: the development and creation of innovative polymeric materials, structural modification of existing polymers, and the formulation of polymer blends that exploit synergistic interactions between constituent components [1]. Polymer modification represents a strategic approach to enhancing the inherent properties of polymeric materials. In particular, modified furan-based polyesters present significant potential for advancing next-generation plastics, especially in the transition from fossil-derived consumer products to biodegradable and biocompatible alternatives. Current research efforts are predominantly centered on the copolymerization, blending, and development of composites based primarily on poly(ethylene 2,5-furandicarboxylate) (PEF), aiming to provide sustainable substitutes within the traditionally petroleum-based plastics market [1,2]. Copolymerization is generally more effective than blending for creating novel materials because it ensures the molecular-level integration of different monomer units, resulting in more uniform properties and improved compatibility. In contrast, blending often leads to phase separation and heterogeneous behavior. In turn, polymer blends are systems composed of two or more polymers combined to yield a material with modified and often enhanced properties. The formulation of such blends is generally more cost-effective and technically straightforward compared to other material modification strategies. Consequently, the blending of PEF and other furan-based polyesters, such as poly(butylene 2,5-furandicarboxylate) (PBF), with both synthetic and natural polymers is gaining increasing attention as a promising route for developing advanced functional materials across diverse application domains [1,2,3].

Thermoplastic polymers can be combined through solution blending, wherein the polymers are dissolved in a single solvent or a mixture of compatible solvents, followed by precipitation and recovery of the resulting blend. The miscibility of these blends, classified as either miscible or immiscible, depends mainly on the thermodynamic compatibility of the components and the specific conditions employed during the preparation process [1]. For instance, Papageorgiou and coworkers [4] reported poly(ethylene terephthalate) (PET)/PEF blends, demonstrating that high temperatures during melt mixing can increase dynamic homogeneity and transesterification. In another study, in order to improve the mechanical properties and sustainability, Chen et al. [5] investigated the combination of PEF and poly(butylene succinate) (PBS) to improve mechanical properties and sustainability.

To effectively reduce the environmental impact of extensively utilized polyester films, Paszkiewicz et al. [6] blended glycol-modified poly(ethylene terephthalate) (PETG) with PEF. A related investigation into the degradation behavior of environmentally friendly polymer blends also highlights a synergistic enhancement in mechanical performance. The resulting blend exhibited exceptional thermal stability, maintaining structural integrity at temperatures up to 300 °C [7]. Another study comparing thermoplastic blends of PEF and PBF demonstrated that variations in bio-based monomer composition significantly influence the thermophysical properties of the resulting materials. The PBF/PEF blend exhibited a single glass transition temperature, indicative of miscibility between the two components. To avoid transesterification reactions typically induced at elevated processing temperatures, the blend was prepared via solution mixing [8].

In the case of comprehensive investigations into various PBF-based blends, Poulopoulou et al. [9] prepared five different blend series of poly(l-lactic acid) (PLA)/PBF, PET/PBF, poly(propylene terephthalate) (PPT)/PBF, poly(butylene 2,6-naphthalenedicarboxylate) (PBN)/PBF, and polycarbonate (PC)/PBF by dissolving the polyesters in a trifluoroacetic acid/chloroform mixture (1:4 *v*/*v*), followed by coprecipitation through the controlled addition of the polymer solution into an excess of cold methanol. The blends were successfully recovered, and PLA/PBF, PBN/PBF, and PC/PBF blends were found to be immiscible. PET/PBF blends exhibited a single, composition-dependent glass transition temperature (T_g_) across all blend formulations, indicating a high degree of miscibility between the components. In another study conducted by this research group, Poulopoulou et al. [10] obtained full biobased PEF/PBF blends, which showed partial miscibility, and the miscibility increased with reactive blending. It was observed that PEF exhibited an accelerated crystallization rate when blended with PBF compared to its neat form, a phenomenon that may offer advantages for the industrial scalability of PEF. Furthermore, Long et al. [11] demonstrated the feasibility of producing fully bio-based PBF/PLA blends via melt mixing. These binary bio-based systems displayed high reflectivity across various processing techniques, including hot pressing, injection molding, and 3D printing.

Natural rubber (NR), a naturally occurring polymer, has been utilized globally since the Industrial Revolution. Its widespread demand across various industries is attributed to its broad applicability in everyday products. However, the intrinsic properties of NR are often inadequate for many practical applications. Consequently, extensive efforts have been devoted to modifying and processing NR to enhance its performance characteristics, thereby making it more suitable for functional use in diverse technological and industrial contexts [12]. In turn, epoxidized natural rubber (epNR) is a renewable, soft, flexible, biocompatible, and biodegradable material. Compared to unmodified natural rubber, epNR contains oxirane rings, which impart high polarity to the polymer chains [13], thereby enhancing its resistance to a variety of chemicals and oils. It is synthesized through the epoxidation of natural rubber, introducing epoxide functional groups at the sites of carbon–carbon double bonds. These modifications are evidenced by characteristic epoxy absorption bands at 836 and 870 cm^−1^ in FTIR spectra [14,15]. Due to the presence of reactive epoxy groups, epNR can participate in further chemical interactions with other natural or synthetic materials, enabling the tailoring of its properties for specific applications. Its distinctive combination of toughness, biocompatibility, and biodegradability, along with its renewable origin, makes it an ideal candidate for enhancing the properties of brittle thermoplastic polyesters such as PLA or furan-based polyesters. While several studies have already been reported on the toughening of PLA with natural rubber (NR), including [16,17,18,19] among others, and despite numerous studies on PEF- and PBF-based blends (as described above) and the proven positive effect of epNR addition on the properties of bio-based polyesters, no reports on PEF/epNR or PBF/epNR blends have appeared in the literature to date.

This study aims to develop and characterize environmentally friendly polymer blends based on bio-based poly(ethylene 2,5-furanoate) (PEF) or poly(butylene 2,5-furanoate) (PBF) with epoxidized natural rubber (epNR). The research focuses on evaluating the miscibility, chemical interactions, thermal behavior, mechanical properties, and oxidative stability of the resulting blends to assess their potential for sustainable applications.

## 2. Materials and Methods

### 2.1. Materials

**Figure 1:**
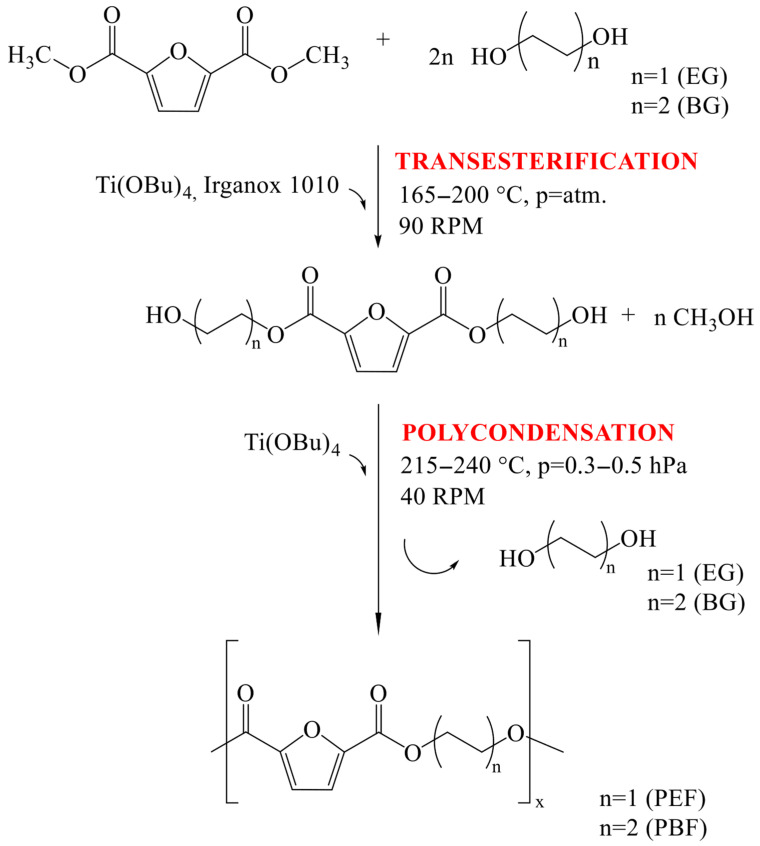
Scheme of the synthesis of PEF and PBF homopolymers.

Homopolymers of poly(ethylene 2,5-furanoate) (PEF) and poly(butylene 2,5-furanoate) (PBF) were synthesized by a two-stage melt polycondensation process (Figure 1). The synthesis setup comprises a 1 dm^3^ steel reactor fitted with a condenser, stirrer, gas inlet, and vacuum pump. To the reactor, a measured amount of substrates is introduced, including dimethyl furan-2,5-dicarboxylate (DMFDC, 99%, obtained from Henan Coreychem Co., Ltd., Zhengzhou City, Henan Province, China), and either 1,2-ethylene glycol (ED, BioUltra, ≥99.5% (GC), from Merck Life Science sp. z o.o., Poznań, Poland) for PEF or 1,4-butylene glycol (BD, Alfa Aesar, 99%, from ThermoFischer GmbH, Kandel, Germany) for PBF, in a molar ratio of diester to diol of 1:2. The initial portion of the catalyst (tetrabutyl orthotitanate, Ti(OBu)_4_ from Fluka Chemie GmbH, Buchs, Switzerland) and the antioxidant (Irganox 1010, from Ciba—Geigy, Basel, Switzerland) are also added. The reactor feed to obtain PEF was as follows: 300 g of DMFDC, 252.7 g of ED, 2.70 g of Irganox, and 0.62 g of the catalyst. The reactor feed required to obtain PBF includes 300 g of DMFDC, 366.7 g of BD, 2.76 g of Irganox, and 0.70 g of catalyst. The reactor is then heated to 165 °C. As the transesterification between DMFDC and diol begins—indicated by methanol by-product dripping—the reaction mixture is progressively heated to a temperature range of 200–215 °C. Once the distilled methanol reaches 90% of its theoretical yield (calculated stoichiometrically)—this stage (transesterification) takes ca. 2 h—the second portion of the catalyst (also Ti(OBu)_4_) is introduced to initiate the second stage, i.e., melt polycondensation. During this stage, the temperature is gradually increased to 240 °C while reducing the pressure to approximately 25–30 Pa. The progress of polycondensation is monitored by observing the increase in stirrer torque, which lasts about 2 h. Upon completion, the resulting homopolymer is extruded from the reactor under nitrogen pressure, cooled to room temperature in a water bath, and subsequently granulated.

Epoxidized natural rubber (Epoxyprene 50) was provided by RESINEX, High Wycombe, UK (Mooney viscosity = 70–100; epoxidation = 50 ± 2).

**Figure 2:**
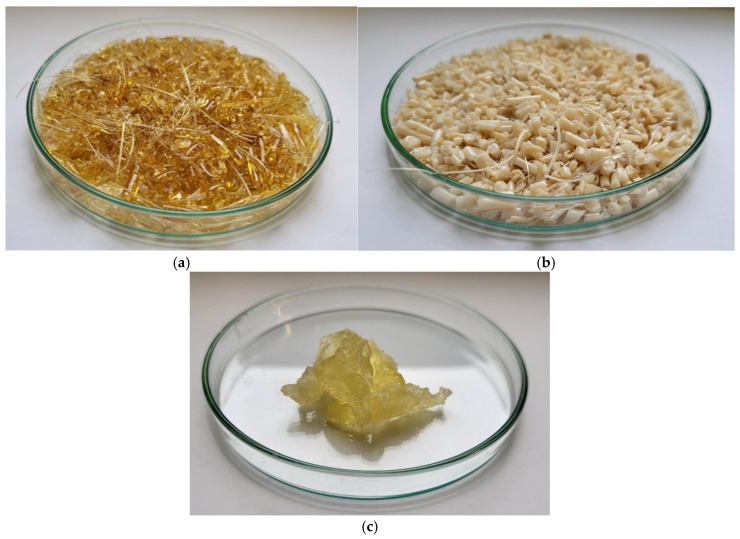
Photographs of synthesized PEF (**a**) and PBF (**b**), and epoxidized natural rubber (**c**).

The obtained homopolymers and epNR, which were used to prepare the PEF/epNR and PBF/epNR blends, are presented in Figure 2.

### 2.2. Preparation of Polyester/epNR Blends

**Figure 3:**
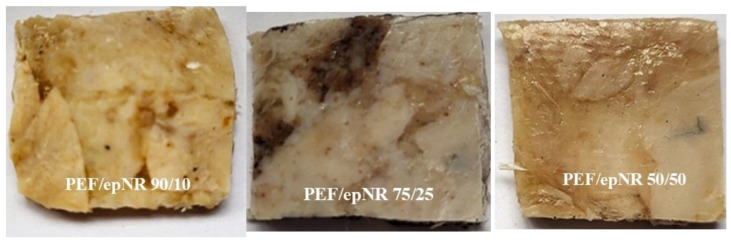

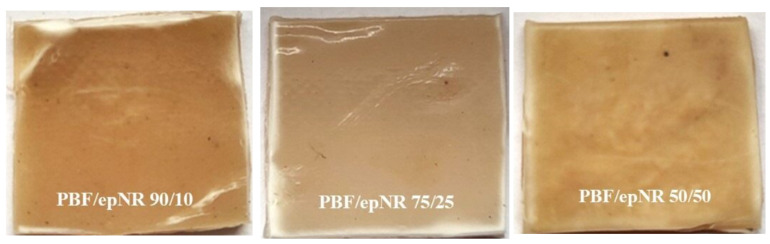
The photographs of the pressed plates.

Compositions (blends) based on thermoplastic polyester (PEF or PBF) and epoxidized natural rubber (epNR), as shown in Figure 3, were prepared with component ratios of 90/10, 75/25, and 50/50 wt./wt. Prior to the mixing process, the samples were dried in a laboratory vacuum dryer (Binder ED115, Tuttlingen, Germany) at 60 °C for 24 h. The melt mixing process was carried out using a Brabender mixer (Plasti-Corder Model PL2100, Brabender GmbH & Co KG, Duisburg, Germany) at 185 °C for PBF-based compositions and 210 °C for PEF-based compositions. The mixing time for all blends was 5 min.

### 2.3. Sample Preparation

The testing samples were prepared using a hydraulic laboratory press (P 200 E, Dr. Collin GmbH, Maitenbeth, Germany). The obtained plates had dimensions of 100 × 100 × 4 mm, and their appearance is presented in Figure 3. The processing parameters are collectively showed in Table 1.

From the obtained plates, samples for strength tests (type A3 sample) were prepared using a Collin P200P hydraulic press (Maitenbeth, Germany) and a standardized die compliant with PN-EN 62811-2015 [20]. The sample preparation process took place in controlled laboratory conditions. The samples were formed at room temperature, using a pressure of 2 MPa for 30 s. Samples used to assess cross-link density in toluene were taken from dumbbell-shaped specimens and prepared as rectangular pieces measuring 7 × 7 × 2 mm. The same samples were used to perform DSC and FTIR analyses. All reference materials, PEF and PBF granules, and as-received epNR raw material, were used for spectroscopic and thermal analyses.

### 2.4. Characterization Methods

The chemical composition of PBF- and PEF-based manufactured compositions was analyzed using a Bruker Tensor 27 Fourier transform infrared (FTIR) spectrophotometer (Billerica, MA, USA), equipped with an attenuated total reflectance (ATR) measuring system. Each sample underwent 32 scans at a resolution of 2 cm^−1^, covering the spectral range of 4000–400 cm^−1^.

Microscopic observations were carried out using a 4K digital microscope VHX-7000 (Keyence, Osaka, Japan).

Thermal properties of polymeric compositions were assessed by differential scanning calorimetry (DSC). The DSC measurements were realized using a DSC 204 F1 Phoenix apparatus (Netzsch, Selb, Germany). Samples 10 ± 0.5 mg in weight were sealed in aluminum pans with a pierced pan. For the PBF/epNR series, the temperature range spanned from −85 °C to 250 °C, while for the PEF/epNR series, it extended from −85 °C to 300 °C. Both series of blends were subjected to heating and cooling rates of 10 °C/min. Measurements were performed under an inert (nitrogen) atmosphere with a 20 mL/min flow rate. The measurement protocol took into account a double heating and cooling cycle under defined conditions. Thermal transition temperatures, including glass transition, cold crystallization (when observable), and melting, were determined from the second heating cycle. Specifically, crystallization (T_c_), cold crystallization (T_cc_), and melting (T_m_) temperatures were identified at the peaks of the respective exothermic and endothermic events.

The thermal stability of the samples was investigated using thermogravimetric analysis (TGA) performed on a Netzsch TG209 F1 instrument (Netzsch, Selb, Germany). Approximately 85 μL of each sample was placed in alumina (Al_2_O_3_) crucibles and subjected to a controlled heating program from 30 °C to 700 °C at a constant heating rate of 10 °C/min. The measurements were carried out under a nitrogen atmosphere with a flow rate of 50 mL/min to prevent oxidative degradation. The first derivative of the thermogravimetric (TG) curves (dTG) was calculated using the instrument’s dedicated analysis software (Proteus, version 8.0, Netzsch, Selb, Germany) to better identify the decomposition steps. The oxidation resistance of the manufactured materials was determined as oxidation onset temperature (OOT) by means of a DSC 204 F1 Phoenix apparatus (Netzsch, Selb, Germany). Measurements were realized under an oxidizing air atmosphere at a heating rate of 10 °C/min, with a temperature range of 20 to 250 °C. The measurements were performed using open aluminum crucibles.

The intrinsic viscosity number (LVN) of the composition series was measured at 30 °C using a phenol/1,1,2,2-tetrachloroethane solvent mixture in a 60:40 weight ratio. The polymer solution had a concentration of 0.5 g/dL, and the measurements were performed with a capillary Ubbelohde viscometer (type Ic, K = 0.03294). The specific weight of applied filler and resulting composites was determined using Ultrapyc 5000 Foam gas pycnometer from Anton Paar (Warsaw, Poland). The following measurement settings were applied: gas—nitrogen; target pressure—5.0 psi; temperature control—on; target temperature—20.0 °C; cell size—small, 10 cm^3^; number of runs—15.

The melt flow rate (MFR) and melt volume rate (MVR) values were determined using a load plastometer (MELT FLOW T.Q., CEAST 6841/048) operating at a specified, constant temperature with an accuracy of ±0.5 °C and a nozzle diameter of 2.095 mm. Measurements were realized according to a standardized procedure described in ISO 1133 [21]. The processing temperature of the composition in the Brabender mixer was selected as the measurement temperature, i.e., 185 °C for PBF-based compositions and 210 °C for PEF-based compositions. The MFR and MVR values were determined at different loads, from 2.16 kg to 10 kg, depending on the material viscosity. The MFR expressed in g/10 min was determined according to Equation (1):

(1) $$MFR\left(T,\;m_{nom}\right)=\frac{600*m}{t}$$

where T—measurement temperature (°C), m_nom_—nominal load (kg), m—average mass of extrudate (g), t—cutting time interval (s), and 600—factor for converting grams per second to grams per 10 min.

The MVR expressed in cm/10 min was calculated using the formula in Equation (2):

(2) $$MVR\left(T,\;m_{nom}\right)=\frac{A*600*l}{t}$$

where T—measurement temperature (°C), m_nom_—nominal load (kg), A—arithmetic mean of the cylinder cross-section area and piston head in square centimeters (nominal value: 0.711 cm, but after taking into account the permissible cylinder diameter tolerances, the product A × 600 can be given as 427), t—previously determined measurement time (s), and l—arithmetic mean of the results of individual distance measurements (cm).

The material’s mechanical performance was evaluated on the basis of a tensile test experiment, according to the PN-EN ISO 527 standard [22], using dumbbell-shaped specimens (type A3) with a gauge length of 20 mm. The measurement was carried out using a universal testing machine Autograph AG-X plus (Shimadzu, Kyoto, Japan), equipped with a 1 kN Shimadzu load cell, a contact optical extensometer, and TRAPEZIUM X software (version 1.4.5, Shimadzu, Kyoto, Japan). The measurements were performed at a 5 mm/min constant crosshead speed, at room temperature and 30% relative humidity. The values of Young’s modulus (E), tensile strength at break (σ_b_), and elongation at break (ε_b_) were determined. At least five samples taken from each series were measured, and the mean value was calculated from the obtained results.

The samples’ hardness was investigated using a Zwick 3100 Shore D durometer (Zwick GmbH, Ulm, Germany). The hardness test was performed according to ISO 48-4:2018/ASTM D2240 [23]. The reported values are the average of ten independent measurements for each material series.

**Figure 4:**
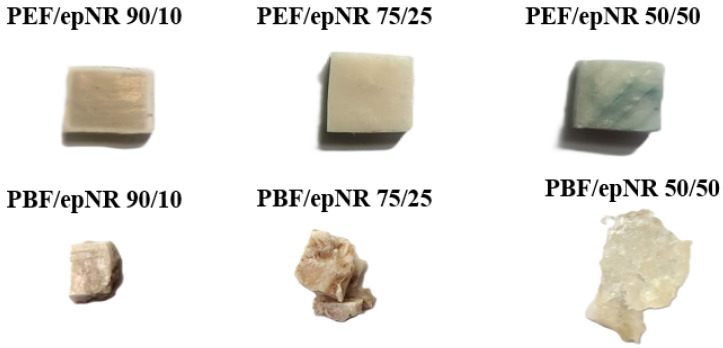
Samples after reaching the equilibrium state during the swelling test.

The apparent cross-link density of each polyester/epNR blend was determined by solvent swelling in toluene at 20 °C. Rectangular specimens (7 × 7 × 2 mm) were precisely cut and weighed before immersion. Swelling was monitored by measuring the sample mass after immersion for 1, 2, 3, 4, 24, 48, and 72 h, until swelling equilibrium was reached. Prior to each weighing, residual toluene was carefully removed from the sample surfaces using a lint-free cloth to ensure accurate measurements. Representative images of the samples at equilibrium swelling are presented in Figure 4. The cross-link density was calculated according to Equation (3) [13], yielding a dimensionless value.

(3) $$V_{r}=\frac{1}{1+\left(\frac{m_{1}}{m_{2}}-1\right)\cdot \frac{\varrho_{k}}{\lambda \cdot \varrho_{t}}}\;$$

where *V*_r_—apparent cross-link density, *m*_1_—mass of the sample before reaching the swelling equilibrium state, *m*_2_—sample mass after reaching the swelling equilibrium state, ρ_t_—toluene density (0.865 g/cm^3^), ρ_k_—epNR density (1.25 g/cm^3^), and λ—epNR mass content in the thermoplastic vulcanizate.

## 3. Results and Discussion

### 3.1. Solubility Assessment and Morphology

Thermodynamic miscibility of polymers can be estimated by the difference in their solubility parameters [24]. Due to significant differences in phase transition temperatures, polymers may exhibit phase solubility or separation. Upon cooling, polymer blends typically form a multiphase structure resulting from micro- and nanophase separation, indicating thermodynamic immiscibility [25,26]. Polymers with similar chemical structures are more likely to dissolve in each other. Notably, compatible or miscible multiphase blends can still display good functional properties. Various methods for estimating the solubility parameter have been proposed by numerous researchers [27].

The Hoy solubility parameter method is a simple way to estimate the miscibility or solubility of one organic material with another (e.g., solvents or polymers) [27]. It is commonly used for analyzing structural features like cis/trans configurations, aromatic substitution patterns (ortho-, meta-, para), and branching [28]. The method predicts the solubility parameter based on three contributions: dispersion forces (δ_d_) (non-polar), polar interactions (δ_p_) (dipole forces), and hydrogen bonding (δ_h_) (present when hydrogen bonds or donor–acceptor interactions occur). Hoy’s system includes four additive molar functions, auxiliary equations, and final expressions for each component, treated as vectors in three-dimensional space, which define the total solubility parameter (δ_tot_) and are calculated following Equation (4) [27]:

(4) $$\delta_{tot}^{2}=\delta_{d}^{2}+\delta_{p}^{2}+\delta_{h}^{2}$$

The complete equation used to determine the solubility of amorphous polymers (P_i,j_) is as follows [27]:

(5) ∆δ=[(δd,Pi−δd,Pj)2+[(δp,Pi−δp,Pj)2+[(δh,Pi−δh,Pj)2]12

Here, Δδ represents the difference in solubility parameters of polymer pairs, with δ_d_, δ_p,_ and δ_h_ corresponding to dispersion forces, polar interactions, and hydrogen bonding, respectively. A smaller difference Δδ < 5 MPa^1/2^ indicates higher solubility [27]. Theoretical solubility parameters for polymer compositions can be calculated.

**Figure 5:**
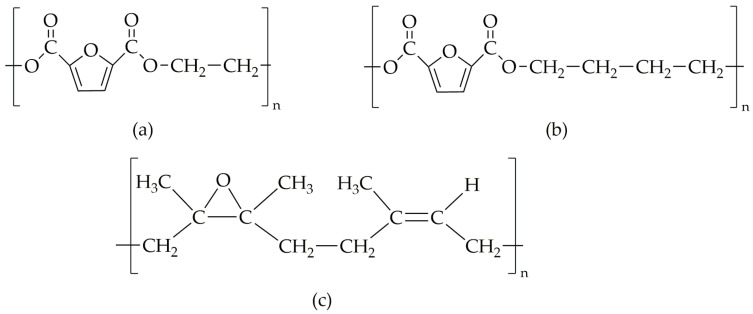
Molecular structures of (**a**) PEF, (**b**) PBF, and (**c**) epoxidized natural rubber.

The molecular structures of homopolymers PEF and PBF and epoxidized natural rubber are shown in Figure 5a–c, respectively, and the total solubility parameters and their components were calculated by using Hoy’s method (Table 2).

Calculations using Hoy’s method indicate that both series of the prepared polymer compositions exhibit higher Δδ parameter values than those characteristic of completely miscible materials (Δδ > 5MPa^1/2^). This confirms that both series of compositions, i.e., PEF/epNR and PBF/epNR, are immiscible and undergo phase separation. However, it is clearly observed that PBF exhibits lower immiscibility with epNR compared to PEF. The increased chain flexibility in PBF enables better conformational compatibility with the flexible, amorphous regions of epNR, thereby improving intermolecular entanglement and miscibility [29]. Moreover, even though both PEF and PBF contain ester and furan functionalities capable of hydrogen bonding, PBF’s longer methylene chain may allow better spatial orientation for interaction with the epoxide groups in ENR, thus enhancing miscibility.

**Figure 6:**
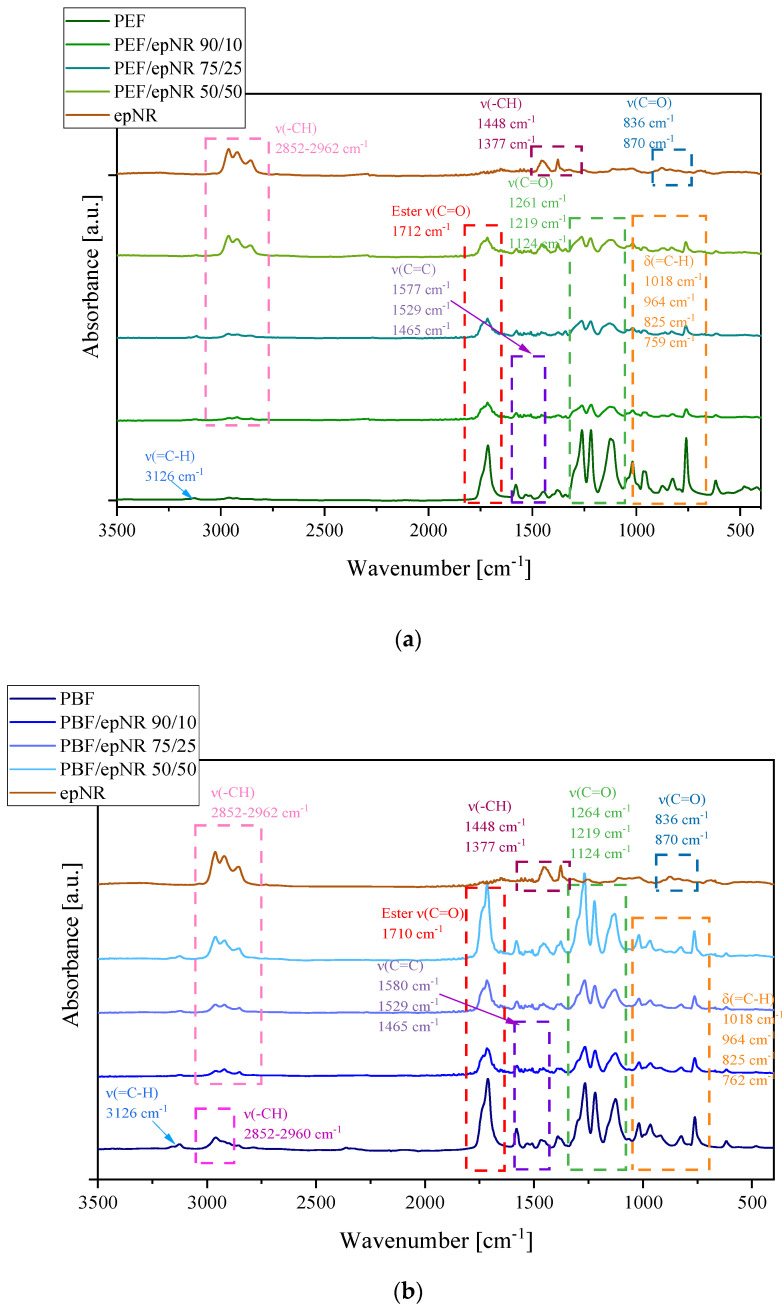
FTIR spectra of neat PEF and PEF/epNR compositions (**a**) and neat PBF and PBF/epNR compositions (**b**).

To study the molecular structure and interfacial interactions between the components of PEF/epNR and PBF/epNR compositions, FT-IR spectroscopy was carried out. The characteristic FT-IR spectra of neat polymers and PEF/epNR and PBF/epNR blends are provided in Figure 6. The FT-IR spectra of both homopolymers reveal a weak absorption band at 3126 cm^−1^, which corresponds to the C–H stretching vibrations characteristic of the furan ring structure present in both polyesters molecules [30,31]. The stretching vibrations of C=O and C-O in the ester group appeared at 1712 and 1261 cm^−1^ in PEF and 1710 and 1264 cm^−1^ in PBF, respectively. The absorption bands observed in the wavenumber range of 2962–2852 cm^−1^ in the PBF spectrum were attributed to the vibration of the C-H bond in the CH_2_ group. Furthermore, for both homopolymers, absorption bands at ca. 1577 cm^−1^ due to the stretching vibration of C=C, at 1015 cm^−1^ due to the breathing vibration of furan ring, and several bands ascribed to the out-of-plane deformation of the 2,5-disubstituted furan heterocycle are observed at approximately 960 cm^−1^, 825 cm^−1^, and 760 cm^−1^ [30,32]. Moreover, for both homopolymers (PEF and PBF), no hydroxyl-terminated absorption peak near 3400 cm^−1^ was observed, which indicated that the polycondensation reactions were complete [30].

While comparing blends’ spectra, one may note that along with the decrease in biopolyesters content, the absorption bands corresponding to stretching vibrations of C=O in the ester group (~1712 cm^−1^), C-O-C stretching (~1260 cm^−1^), and =C-O-C= (~1218 cm^−1^, ~760 cm^−1^) ring vibrations became less intense. In turn, one can clearly observe the characteristic absorption bands of epNR at 2854, 2917, and 2961 cm^−1^, due to C–H stretching and −CH_2_ groups, respectively, which exhibit a decreasing intensity with lowered epNR content in blends [14,15,33,34]. Additionally, epNR exhibited weak symmetric and asymmetric epoxide bands, with C=CH wagging at 836 cm^−1^ and C–O–C stretching associated with partial ring opening of the epoxide group at 870 cm^−1^ [33]. Moreover, only in epNR and blends with the highest epNR content (PEF/epNR 90/10 and PBF/epNR 90/10), absorption bands at 1377 and 1448 cm^−1^, attributed to the stretching of the −CH_3_ and C–H groups, were visible [14,15].

**Figure 7:**
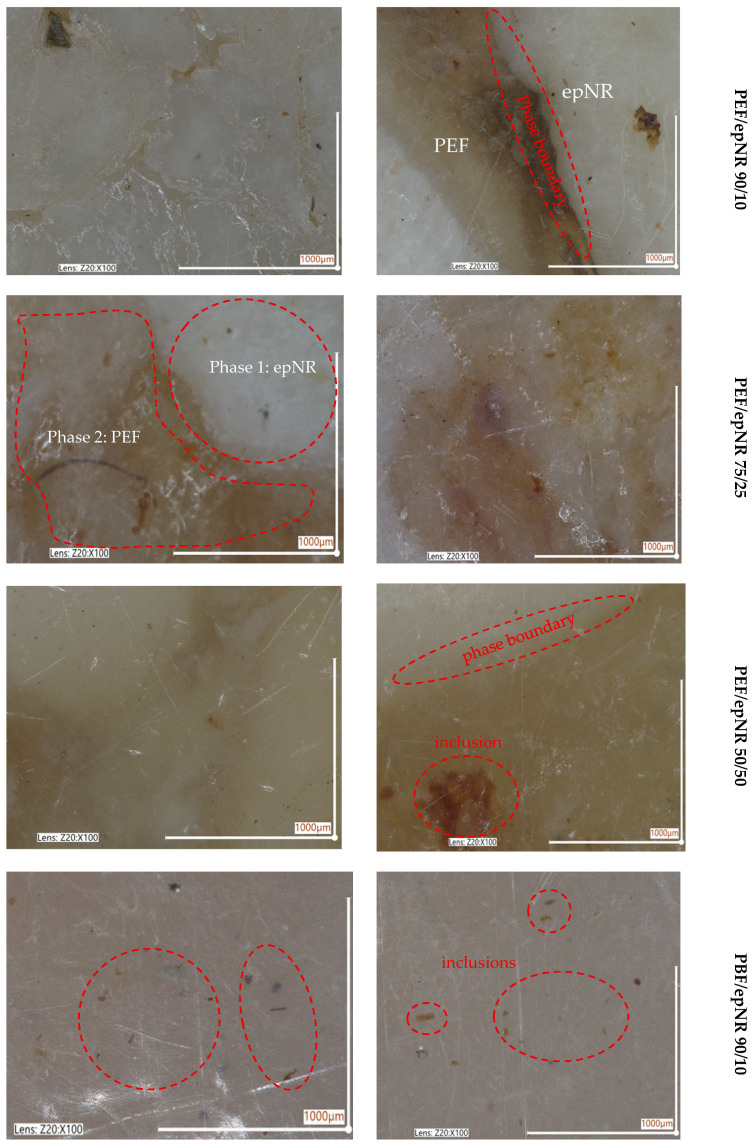

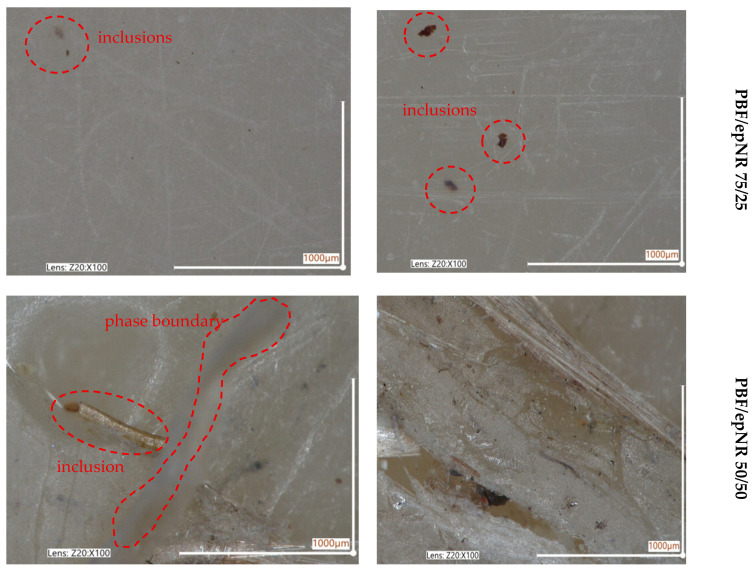
Optical images of prepared PEF-based and PBF-based compositions.

Furthermore, the obtained PBF- and PEF-based composites containing various amounts of epoxidized natural rubber were subjected to microscopic analysis. The aim was to compare the surface structure of the materials and assess the impact of epNR addition on the homogeneity and occurrence of defects within the composition. Two images were taken for each material using an optical microscope at 100× magnification. Microscopical images of both series of polyester-based compositions are presented in Figure 7. The PEF/epNR 90/10 system exhibits an irregular surface with numerous defects, including epNR inclusions and microcracks. The occurrence of these defects suggests that achieving homogeneous compositions based on PEF and epNR was not possible. Increasing the content of epNR in PEF resulted in even greater issues with homogeneity and the presence of defects, such as inclusions and microcracks (marked in the micrographs with red circles and arrows). Additionally, observed changes in surface color may indicate uneven distribution of epoxidized rubber during mixing within the PEF matrix. The highest number of defects among all tested samples was observed in the system containing 50% of epNR. Pronounced inclusions and extensive cracking indicate significant issues with blend homogeneity, while the noticeable surface color disparity further confirms insufficient mixing between PEF and the epoxidized rubber. Although epNR is expected to promote good interfacial compatibility between the blend components, particularly given its potential function as a dual compatibilizer that significantly enhances interfacial adhesion [35], in the case of this series of compositions, the application of an additional compatibilizer between the components would be justified.

Numerous efforts to improve compatibility between PEF and epNR, as well as with other biobased polyesters, have been described in the literature [36,37,38,39]. Fredi et al. [38] investigated the properties of melt-mixed PEF/PLA blends as a function of the PEF weight fraction (1–30 wt.%) and the amount of the commercial compatibilizer/chain extender Joncryl ADR 4468, which mitigated the immiscibility of the two polymer phases by decreasing and homogenizing the PEF domain size. The synergistic effects of PEF and compatibilizer/chain extender in improving the thermal, mechanical, UV-shielding, and gas-barrier properties of PLA were emphasized. In turn, Ahmed et al. [39] provided a comprehensive report on the development of blends of biorenewable PEF with synthetic polyolefins. The authors claimed that owing to the intrinsic immiscibility between PEF and polyolefins, two conventional compatibilizers (styrene-ethylene/butylene-styrene-graft-maleic anhydride (SEBS-g-MA) and polyethylene-graft-maleic anhydride (PE-g-MA)) were employed to enhance interfacial adhesion between the polyester and polyethylene phases. The type and concentration of the compatibilizers significantly influenced the resulting phase morphology within the polymer blends. The interaction between the anhydride functionalities of the compatibilizers and the terminal hydroxyl groups of the polyester phase played a key role in altering the morphology and influencing the physical properties of the resulting blends. However, in both cited solutions, commercially available compatibilizers were used, whereas our research focuses on the possibility of directly connecting biopolyesters with epNR. In the next stage, the study will concentrate on the use of dedicated compatibilizers with specific functional groups for the system based on furan thermoplastic polyester. In the case of the PBF/epNR 90/10 system, however, we observe a relatively smooth and uniform surface, with visible minor micro-damages and delicate scratches and a minimal amount of impurities. The high PBF content makes the structure more compact and uniform. The PBF/epNR 75/25 sample is slightly more diverse than in the case of the 90/10 proportion, with visible, delicate depressions and minor inclusions. In the case of the PBF/epNR 50/50 sample, a clearly less uniform surface was observed, with visible damage and numerous inclusions. Taking into account our observations and the remarks of other groups [8,9], our further research should focus on the usage of compatibilizers or optimized processing conditions to enhance interfacial adhesion and morphology of blends with the content of epNR > 25%. Investigating the influence of morphology on utilitarian properties could also guide future applications of these sustainable materials.

### 3.2. Thermal Properties

**Figure 8:**
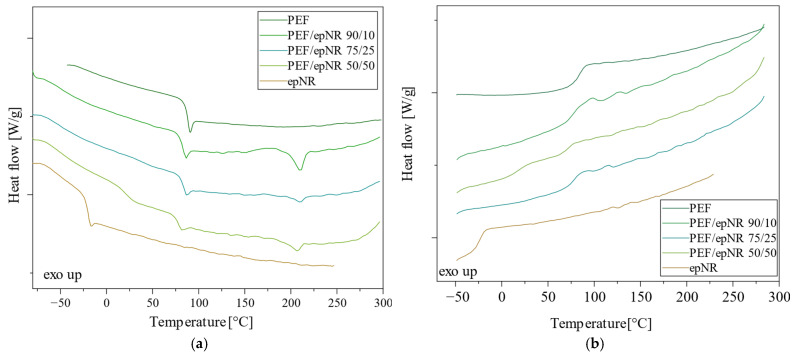
DSC thermograms recorded during the second heating (**a**) and cooling (**b**) for neat PEF and PEF/epNR compositions.

**Figure 9:**
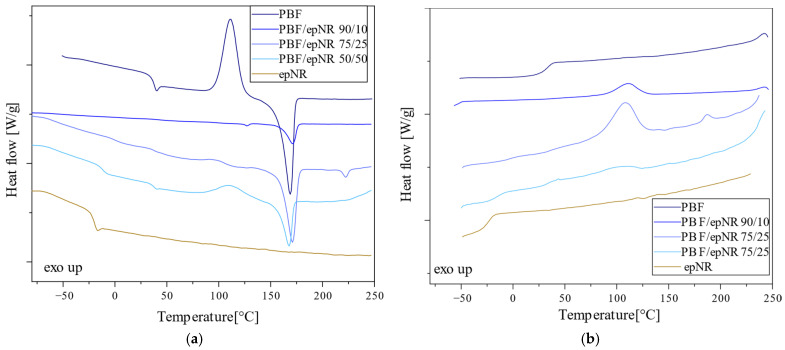
DSC thermograms recorded during the second heating (**a**) and cooling (**b**) for neat PBF and PBF/epNR compositions.

Differential scanning calorimetry (DSC) was used to determine the effect of epNR addition on the characteristic phase transition temperatures and the corresponding heat changes for selected thermoplastic polyesters. The DSC curves recorded during the second heating and cooling for PEF and PEF-based compositions with epNR are presented in Figure 8. Meanwhile, the DSC curves obtained for PBF and PBF-based compositions with epNR are shown in Figure 9. The characteristic phase transition temperatures and their corresponding thermal effects are summarized in Table 3.

Based on the DSC data obtained from the second heating, PEF was identified as an amorphous polymer, exhibiting only a glass transition at 86.7 °C without a melting peak. In turn, epNR displayed a glass transition temperature of around −23 °C. The addition of epNR to PEF resulted in a downward shift in PEF’s glass transition temperature. Furthermore, the DSC curves of PEF/epNR compositions revealed melting peaks. The peak melting temperatures were recorded at 210.2 °C for the composition containing 10 wt.% epNR, 209.7 °C for the composition containing 25 wt.% of epNR, and 207.6 °C for the composition containing 50 wt.% epNR. The incorporation of epNR increased the molecular mobility of PEF chains, facilitating the formation of a crystalline phase, as evidenced by the melting peaks observed during the second heating cycle.

The DSC curves for neat PBF exhibited a glass transition at 36.2 °C, melting at 169 °C, and cold crystallization at 111.4 °C. The incorporation of epNR did not significantly affect the glass transition temperature of PBF but led to a shift in the glass transition temperature of the rubber-rich phase toward lower temperatures. On the DSC curve of the composition containing 25 wt.% epNR, a glass transition associated with the rubber-rich phase was observed. No linear correlation was found between the melting temperature and the composition ratio. Only neat PBF and the composition containing 50 wt.% of epNR exhibited a peak corresponding to cold crystallization. The DSC cooling curves revealed that neat PBF did not crystallize from the melt, whereas the PBF/epNR compositions crystallized from the melt at approximately 109 °C. This may be attributed to disruptions in the crystalline structure of PBF caused by epNR or an uneven distribution of components within the composition.

The oxidation resistance results of PEF, PBF, and their blends with epNR were analyzed to determine the oxidation onset temperature (OOT) using DSC, and the results are listed in Table 3. The description of oxidation resistance processes in furan-based thermoplastics is not widely discussed in the literature. Zubkiewicz et al. [40] compared the thermo-oxidative stability of PBF and PLA, as well as other biopolyesters, using thermogravimetric analysis conducted in an air atmosphere. The materials tested in this method showed comparable temperatures at 10% mass loss (369 °C for PEF; 363 °C for PBF), which were used as an evaluation criterion. Terzopoulou et al. [41] compared the oxidation onset temperature of PEF modified with various stabilizers, and pure PEF in the considered case showed a similar OOT value of 242.9 °C, as reported earlier. For PEF blends, the introduction of epNR, which has significantly lower oxidation resistance, resulted in a decrease in the OOT of the mixtures, with a greater share of elastomeric content. The better compatibility of PBF with epNR resulted in an almost constant OOT value and no dependence of epNR concentration on the intensity of the decrease in thermooxidative stability. It should be emphasized, however, that despite the reduced resistance to oxygen in the molten state, all blends retained a higher OOT value compared to the reference epNR series.

**Figure 10:**
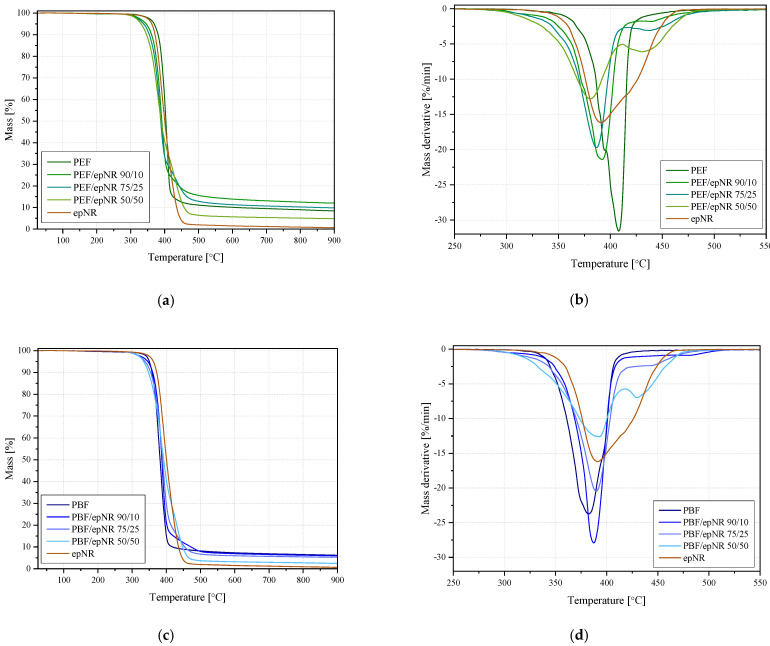
TG (**a**) and DTG (**b**) for PEF/epNR and TG (**c**) and DTG (**d**) for PBF/epNR.

Figure 10 summarizes the TG and DTG curves for PEF, PBF, and their blends with epNR in a narrower temperature range. Detailed data on the characteristic temperature values at 2, 5, and 10% weight loss (T_2%_, T_5%_, T_10%_), together with information on residue at 900 °C and detailed data on the position and intensity of the maxima read from the DTG curves, are presented in Table 4. PBF exhibited single-step decomposition, with the maximum degradation process described by the maximum DTG curve at 382.4 °C, which results from the degradation of the polymer backbone, specifically the cleavage of ester linkages [6]. In the case of epNR, a complex course of degradation and overlapping phenomena can be observed; however, it lacks a specific characteristic peak on the DTG curve. The main broad degradation peak was observed at 391.1 °C, indicating a complex and non-uniform structure of the epoxidized natural rubber. A similar course of the TG curve was reported before [42]. Unmodified PEF and the blends of PBF/epNR and PEF/epNR were determined by a two-step decomposition process. Both DTG peaks for PEF are related to the decomposition of the furanic acid unit [43]. The occurrence of two peaks may be related to the heterogeneous structure of the polymer itself and has been reported in earlier studies [43,44]. The observed lower value of the DTG_1_ maximum for PEF at 394.3 °C should not be related to incomplete polymerization or the presence of impurities at the synthesis level, due to the characteristic values of substrate decomposition and contaminated PEF series reported by Dong et al. [44]. The decomposition temperature for PBF aligns with those reported earlier [45]. The reduction of the onset of thermal degradation of biopolyesters with the addition of epNR, as well as the appearance of the second high-temperature degradation phenomenon for the mixtures of PEF and PBF with epNR, was already observed for another biopolyester (PLA) by Bijarimi et al. [42]. Both PBF and PEF showed quite a significant share of the thermally stable char at 900 °C. The gradual increase in the epNR share led to its subsequent decrease. Considering residue with the highest use of epNR (50/50 series)—lower than would result from a stoichiometric share of epNR—it can be assumed that the system became reactive concerning both components, which the above-mentioned observed changes can also confirm, i.e., earlier decomposition onset and presence of a second degradation step as a result.

### 3.3. Physicochemical, Processing, and Mechanical Properties

**Figure 11:**
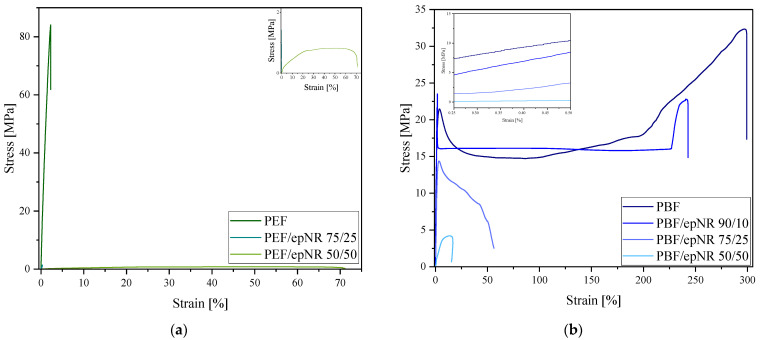
Representative stress–strain curves for PEF-based (**a**) and PBF-based (**b**) compositions.

The physicochemical properties of the obtained materials were evaluated based on measurements of limited viscosity number (LVN) and density. Processing properties were assessed by determination of the melt flow rate (MFR) and melt volume rate (MVR). Mechanical properties were assessed through uniaxial tensile testing and hardness measurements. The determined parameters are summarized in Table 5, while representative stress–strain curves are presented in Figure 11a,b.

The LVN value of the synthesized PEF was determined to be 0.426 dL/g. This value is comparable to a previously reported LVN of 0.503 dL/g [6] and is significantly higher than the LVN values typically obtained for PEF synthesized via solid-state polymerization (SSP) [46]. The incorporation of epNR had a nonlinear effect on the LVN of the PEF-based compositions. The addition of 10 wt.% epNR led to a decrease in LVN, whereas increasing the epNR content resulted in a systematic increase in LVN, from 0.426 dL/g for the 25 wt.% epNR composition to 0.463 dL/g for the compositions containing 50 wt.% epNR. The PBF homopolymer exhibited an intrinsic viscosity of 0.606 dL/g. The addition of 25 wt.% epNR further caused an increase in the LVN to 0.803 dL/g. However, the final composition containing 50 wt.% epNR showed a reduced IV, which may be attributed to partial degradation of the material during processing.

The values of density for the synthesized homopolyesters were 1.40 g/cm^3^ for PEF and 1.34 g/cm^3^ for PBF, respectively. A practically linear decrease in density values was observed with increasing epNR content in both series of compositions. This decrease in the density can be attributed to several factors, foremostly from the lower intrinsic density of epNR 1.25 g/cm^3^, compared to biopolyesters, which decreases the overall density of the system proportionally. However, since the blends of 50/50 exhibited even lower values of density than both of the components individually, such a decrease might be due to the limited miscibility between epNR and the furan-based polyesters, while the resulting morphology (Figure 7) includes phase-separated structures with voids and weak interfacial adhesion. These microstructural features most probably reduce the packing efficiency of the polymer chains, further lowering the apparent or measured density. Moreover, the molecular flexibility and rubbery behavior/nature of epNR might have introduced additional free volume into the compositions, which decreases the overall density.

The analysis of melt flow indexes (MFR and MVR) conducted for homopolymers and their blends with varying amounts of epoxidized natural rubber enabled the evaluation of the modifier’s influence on the flow behavior under load, which is a parameter most often used in industry for determining the processing properties of thermoplastics. The results indicate that the addition of epNR significantly affects the MFI values, which may be attributed to alterations in the supramolecular structure and changes in the material’s flow behavior under applied load and elevated temperature. For PEF under a load of 2.16 kg, the MFR value was 20.4 g/10 min, and the MVR was 22.2 cm^3^/10 min. When the load increased to 5 kg, the MFR decreased to 18.7 g/10 min, while the MVR increased to 49.8 cm^3^/10 min. The addition of 10 wt.% epNR to the PEF composition resulted in a significant reduction in flowability, with flow occurring only at a load of 21 kg. Moreover, as the epNR content increased, a probable cross-linking phenomenon between the polyester and the epNR was observed, preventing measurement even at the highest load (21 kg). This cross-linking of PEF in the blend with epNR during the MFR/MVR test was likely caused by reactions between the epoxide groups and the terminal groups of PEF, as well as the test conditions, i.e., elevated temperature and prolonged residence time in the molten state, leading to the formation of a spatially cross-linked network that restricts flow.

In the case of the homopolymer PBF, the influence of epNR was more pronounced. PBF exhibited MFR and MVR values of 6.0 g/10 min and 6.7 cm^3^/10 min, respectively, under a load of 2.16 kg. Increasing the load to 5 kg resulted in an increase in these values to 14.9 g/10 min for MFR and 16.4 cm^3^/10 min for MVR, while at 10 kg, the values reached 38.7 g/10 min (MFR) and 35.8 cm^3^/10 min (MVR). The addition of epNR to PBF at a 90/10 ratio caused a significant decrease in both MFR and MVR values, indicating increased viscosity of the blend. Specifically, for the PBF 90/10 composition at 2.16 kg load, a reduction in MFR by approximately 42% and in MVR by approximately 49% was observed compared to the PBF homopolymer. However, at a 5 kg load, values of both MFR and MVR nearly doubled relative to the reference sample (homopolymer PBF). Further increase in the epNR content led to decreases in MFR and MVR values, confirming that epNR significantly affects the flow behavior of PBF by increasing viscosity and reducing flowability.

The PEF homopolymer exhibited the highest Young’s modulus of 5.43 GPa and a relatively high tensile strength of 84.1 MPa, but very low elongation at break of only 2.3%. This mechanical profile indicates a rigid and brittle character, lacking the ability to undergo significant deformation prior to failure. Due to insufficient sample quantity (resulting from the impossibility of cutting out dumbbell-shaped samples from compressed plates), tensile testing could not be conducted for the PEF/epNR 90/10 composition. The incorporation of 25 wt.% epNR into PEF led to a substantial reduction in stiffness, with the maximum stress decreasing by approximately 98%. A similarly drastic decrease was observed in elongation at break, which dropped by about 95% compared to the reference sample. For the PEF/epNR 50/50 system, a further reduction in maximum stress (around 40% lower than that of the PEF/epNR 75/25 composition) was noted. However, the elongation at break increased to approximately 70%, indicating an enhanced ability to undergo plastic deformation. Increasing the epNR content to 50 wt.% significantly reduced the material’s stiffness while improving its elasticity, rendering it more ductile and susceptible to deformation.

The synthesized PBF is characterized by a Young’s modulus of 2.35 GPa and a tensile strength of 50.0 MPa. These mechanical properties are comparable to those previously reported for PBF annealed for 1 h at 100 °C [40]. However, the high elongation at break of 291.5% indicates significant flexibility and an excellent capacity for plastic deformation. The incorporation of epNR into PBF results in a reduction in both tensile strength and stiffness. Compared to the reference sample, the composition containing 10 wt.% epNR exhibits a decrease in tensile strength of approximately 50%, and a 17% reduction in elongation at break. This declining trend becomes more pronounced with higher epNR content. The sample containing 50 wt.% epNR shows an approximate 90% reduction in tensile strength and a dramatic 95% decrease in elongation at break relative to the PBF homopolymer. It was concluded that the addition of epNR to PBF systematically deteriorates its mechanical performance, resulting from insufficient interactions between components in the compositions.

The hardness of the materials was measured using the Shore D method, and the results are presented in Table 4. The addition of epNR in various proportions (90/10, 75/25, 50/50) leads to a reduction in Shore D hardness values for both PEF- and PBF-based compositions. This effect becomes particularly pronounced in the compositions with the highest content of epoxidized rubber (50/50), where a decrease of approximately 40% is observed compared to the PEF homopolymer, and about 19% compared to the PBF homopolymer. This decrease in the values of hardness may be attributed to the soft and elastomeric nature of the epNR, which reduces the overall rigidity of the composition. Additionally, increasing the epNR content likely disrupts the polymer matrix continuity, leading to a more flexible and less densely packed structure, which is in line with the observations made above.

Besides, due to the aforementioned potential applications of polyester-based compositions with epNR, the obtained mechanical properties were compared with those of Hytrels, a family of thermoplastic polyester elastomers produced by DuPont. These materials combine the elasticity of rubber with the strength and processability of thermoplastics. For comparative purposes, Hytrel 6356 and Hytrel 4556 were selected, as their hardness is comparable to the tested samples [47,48]. By comparing mechanical parameters such as stiffness, tensile strength, and elongation at break, it can be concluded that Hytrel 6356 and 4556 exhibit significantly superior mechanical performance, which is particularly evident when compared to the PEF/epNR 75/25 and PBF/epNR 50/50 compositions, which have similar hardness values. The Hytrel grades selected for comparison exhibited much better strength properties, which result from their specific chemical structure and, most importantly, strong interfacial interactions between the polyester and elastomer phases, interactions that were not observed in the PEF- or PBF-based compositions with epNR. This suggests the relevance of continuing the present research, particularly in exploring the use of a dedicated compatibilizer to enhance interactions between the blend components.

### 3.4. Mass Intake and Apparent Cross-Link Density

**Figure 12:**
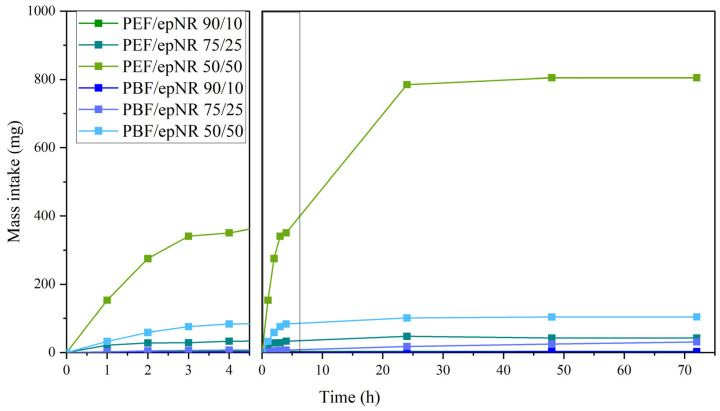
Mass intake over time of PEF/epNR and PBF/epNR compositions immersed in toluene.

Mass intake measurements of toluene for biopolyester-based blends containing epNR were performed to evaluate the interactions between the phases in the compositions. Figure 12 shows the mass intake of samples immersed in toluene, while Table 6 presents the apparent cross-link density values of the analyzed samples, calculated using Equation (3). Mass uptake measurements were recorded over the initial 4 h and subsequently at 24 h intervals for a total duration of 72 h. Analysis of the swelling behavior within the first 4 h revealed a pronounced increase in toluene absorption for samples with higher epNR content, as evidenced by a more rapid and substantial mass gain. The highest mass intake was exhibited by PEF/epNR 50/50, resulting from the insufficient compatibilization and partial solubility of the components between phases, which is in line with the observations made above in the previous sections. Moreover, an increase in cross-link density was observed in two series of blends, along with an increase in epNR. While in systems containing 10 wt.% and 25 wt.% epNR, the cross-link density values are comparable in both series, a significant difference was observed at a 50 wt.% content. In the PBF-based series, the increase in cross-link density was relatively linear, whereas for the PEF/epNR 50/50 composition, this increase was nearly an order of magnitude higher. This phenomenon may be attributed to differences in the chemical structure and reactivity of PEF and PBF toward the epNR. These findings are in agreement with the observations made in our previous study [49], where double-decker silsesquioxanes (DDSQ-eter-4OH) with dedicated functional hydroxyl groups were used as a compatibilizer in the eco-friendly PLA/epNR system. An increase in cross-link density was observed with increasing epNR content up to 30 wt.%. However, higher epNR content led to a decrease in apparent cross-link density. In the 60/40 system, the addition of DDSQ-eter-4OH had no significant effect. The increase at ≤30% epNR resulted from more cross-links or chemical bonds between polymer chains. Beyond this threshold, structural changes occur at 40% epNR, where phase inversion takes place, with epNR becoming the continuous phase and PLA the dispersed one.

## 4. Conclusions and Future Perspectives

This study analyzed the effect of adding epNR on the properties of thermoplastic polyesters derived from renewable sources, namely PEF and PBF. The objective of the research was to determine the conditions and feasibility of obtaining blends based on furan-based polyesters with epNR and to evaluate how their properties change depending on the blend composition. Calculations based on Hoy’s method, which are typically applied to partially immiscible systems, along with FTIR, microscopic, and DSC analyses, suggest the presence of specific interfacial interactions between the polymer components. Additionally, the high processing temperature likely promoted chain entanglement at the interface. These interfacial phenomena contributed to the formation of two bio-based blend series containing epNR, which exhibited properties of potential interest for practical applications. Optical microscopy studies revealed that increasing the content of epNR affects the morphology and uniformity of the composition, leading to the formation of defects at higher concentrations. Compatibility issues between PEF and the epNR were also observed, indicating the need for further research, such as the introduction of compatibilizers. From the DSC analysis, we found that the addition of epNR to PEF lowers the glass transition temperature and promotes partial crystallization, likely due to increased molecular mobility. As the epNR content increased, a gradual decrease in melting temperature was also observed. In the case of PBF, the epNR incorporation had no significant effect on the glass transition temperature or melt crystallization, but it did cause structural disturbances and phase separation, as evidenced by the appearance of a new glass transition in the composition containing 25% of epNR. During cooling, the PBF homopolymer did not crystallize from the melt, in contrast to the PBF compositions containing epoxidized rubber. In turn, the TGA revealed distinct thermal degradation behaviors for homopolyesters and their blends with epNR, with PBF showing single-step decomposition and PEF-based materials displaying a two-step degradation profile, likely due to polymer polydispersity and complex degradation mechanisms. The addition of epNR generally reduced the onset temperature of thermal degradation and contributed to the appearance of a second degradation step, suggesting possible chemical interactions and increased reactivity between the blend components.

Physicochemical studies indicate that the addition of epNR significantly affects the MFI values, which may be attributed to alterations in the supramolecular structure and changes in the material’s flow behavior under applied load and elevated temperature. However, for the series based on PEF, as the epNR content increased, a probable crosslinking phenomenon between the polyester and the epNR was observed, which hindered measurement even at the maximum load of 21 kg. Mechanical testing demonstrated that the addition of epNR to PEF caused an increase in flexibility but decreased stiffness and tensile strength, especially at higher epNR contents. For the PBF-based series, a gradual decrease in tensile strength and modulus was observed with increasing epNR content. The superior mechanical properties for PBF were obtained at a lower epNR content (10%), making this system applicable in uses requiring flexibility and moderate tensile strength, but only where high mechanical strength is not essential. In addition, hardness measurements confirmed that the addition of epNR reduced hardness for both the PEF- and PBF-based series of compositions. Higher epNR contents in the PEF-based series of compositions led to network formation, which hinders processing and confirms compatibility issues between components. Moreover, the toluene mass intake and cross-link density measurements indicate that increasing epNR content significantly affects phase interactions in biopolyester-based blends. Notably, the PEF/epNR 50/50 composition exhibited the highest mass uptake and a pronounced increase in cross-link density, highlighting limited compatibilization and stronger chemical interactions compared to the more linear behavior observed in the PBF-based blends.

The final composition configuration should be selected based on the specific application, taking into account both mechanical performance requirements and processing parameters. Further research should be focused on improving phase compatibility and modifying interactions between components, which would enable enhanced mechanical, physicochemical, and processing properties at higher contents of epNR. The obtained results provide a valuable contribution to the development of elastomers derived from renewable resources, which may have significant implications for the plastics industry and sustainable development.

## Figures and Tables

**Table 1:** Processing parameters used for sample manufacturing.

Stage	Temperature [°C]	Pressure [bar]	Time [s]	
Stage I	205	15	180	Series PEF/ epNR
Stage II	205	20	120	
Stage III	175	20	60	
Stage I	185	15	180	Series PBF/ epNR
Stage II	185	20	120	
Stage III	155	20	60	

**Table 2:** Solubility parameters of the PEF/epNR and PBF/epNR calculated by using Hoy’s method.

Solubility Parameters	epNR [MPa^−1^]	PEF [MPa^−1^]	PBF [MPa^−1^]
δ_tot._	19.54	27.33	25.49
δ_p_	5.45	17.03	15.24
δ_h_	8.88	15.67	13.54
δ_d_	16.53	14.21	15.30
	Δδ_epNR/PEF or PBF_	13.63	10.91

**Table 3:** The values of characteristic phase transition temperatures and corresponding thermal effects.

Sample	T_g1_ (°C)	ΔC_p1_ (J/g°C)	T_g1_ (°C)	ΔC_p1_ (J/g°C)	T_cc_ (°C)	ΔH_cc_ (J/g)	T_m_ (°C)	ΔH_m_ (J/g)	T_c_ (°C)	ΔHc (J/g)	OOT (°C)
epNR	−20.9	0.454	-	-	-	-	-	-	-	-	191.7
PEF	-	-	86.7	0.414	-	-	-	-	-	-	242.9
PEF/epNR 90/10	-	-	82.1	0.251	-	-	210.2	5.14	-	-	238.0
PEF/epNR 75/25	-	-	82.2	0.26	-	-	209.7	1.68	-	-	202.5
PEF/epNR 50/50	−23.1	0.160	77.5	0.261	-	-	207.6	2.23	-	-	198.6
PBF	-	-	36.2	0.312	111.4	39.74	169	33.75	-	-	264.8
PBF/epNR 90/10	-	-	36	0.002	-	-	171.2	9.29	111.6	7.32	211.9
PBF/epNR 75/25	−3.2	0.096	35.5	0.053	-	-	171	34	108.3	28.91	214.4
PBF/epNR 50/50	−23.1	0.160	36.6	0.123	109.5	11.17	167	14.5	110.4	3.67	214.0

T_g_, glass transition temperature; ∆C_p_, change in heat capacity; T_cc_, ∆H_cc_, cold crystallization temperature and the corresponding enthalpy of crystallization; T_m_, ∆H_m_, melting temperature and the corresponding enthalpy of melting; T_c_, ∆H_c_, crystallization temperature and the corresponding enthalpy of crystallization; OOT—oxidation onset temperature.

**Table 4:** Thermal parameters obtained by TGA for PEF/epNR and PBF/epNR.

Sample	T_2%_ (°C)	T_5%_ (°C)	T_10%_ (°C)	Residue (%)	DTG_1_ (°C; %/min)	DTG_2_ (°C; %/min)
epNR	344.8	362.4	371.9	0.63	391.1; −16.19	-
PEF	348.0	369.1	380.7	8.44	394.3; −20.08	408.2; −31.58
PEF/epNR 90/10	319.9	345.7	361.6	12.05	392.1; −21.42	439.6; −1.81
PEF/epNR 75/25	314.2	338.4	354.9	9.84	386.6; −19.73	437.8; −3.09
PEF/epNR 50/50	311.5	330.7	346.8	4.81	380.7; −12.80	430.4; −6.12
PBF	337.9	350.3	358.4	6.2	382.4;−23.75	-
PBF/epNR 90/10	316.9	345.9	360.2	5.94	387.4; −27.93	478.9; −0.90
PBF/epNR 75/25	314.4	338.0	369.0	5.26	389.8; −20.46	442.2; −2.39
PBF/epNR 50/50	316.5	334.0	347.9	2.46	392.6; −12.6	429.9; −7.00

**Table 5:** Physicochemical and mechanical parameters for compositions based on biopolyesters and epNR.

Sample	LVN (dL/g)	d (g/cm^3^)	MFR (g/10 min)	MVR (cm^3^/10 min)	E (GPa)	σ_b_ (MPa)	ε_b_ (%)	H (ShD)
epNR	0.426	-	-	-	5.43 ± 0.03	84.07 ± 4.43	2.27 ± 0.14	-
PEF	0.402	1.403	20.4 ^a^ 18.7 ^b^	22.2 ^a^ 49.8 ^b^	-	-	-	74 ± 1
PEF/epNR 90/10	0.439	1.380	4.2 ^d^	3.7 ^d^	0.14 ± 0.01	1.36 ± 0.07	0.12 ± 0.01	70 ± 1
PEF/epNR 75/25	0.463	1.298	-	-	0.01 ± 0.01	0.82 ± 0.04	71.28 ± 5.47	67 ± 1
PEF/epNR 50/50	0.606	1.171	-	-	2.35 ± 0.12	50.19 ± 0.21	291.55 ± 27.87	44 ± 1
PBF	0.637	1.343	6.0 ^a^ 14.9 ^b^ 38.7 ^c^	6.7 ^a^ 16.4 ^b^ 35.8 ^c^	1.62 ± 0.14	25.01 ± 0.92	274.28 ± 10.18	67 ± 1
PBF/epNR 90/10	0.803	1.274	3.4 ^a^ 11.1 ^b^	3.4 ^a^ 11.7 ^b^	0.51 ± 0.01	13.36 ± 1.27	52.11 ± 3.34	64 ± 1
PBF/epNR 75/25	0.581	1.231	0.8 ^a^ 3.2 ^b^	0.6 ^a^ 3.3 ^b^	0.06 ± 0.01	4.83 ± 0.34	14.98 ± 1.07	62 ± 1
PBF/epNR 50/50	0.426	1.141	0.7 ^b^	0.4 ^b^	5.43 ± 0.03	84.07 ± 4.43	2.27 ± 0.14	64 ± 1

LVN—limited viscosity number; d—density; MFR—melt flow rate and MVR—melt volume rate determined at 205 °C for PEF-based compositions and 185 °C for PBF-based compositions and loads of (a) 2.16 kg, (b) 5 kg, (c) 10 kg, and (d) 21.6 kg.

**Table 6:** The apparent cross-link density of PEF/epNR and PBF/epNR compositions.

Sample	Apparent Cross-Link Density
PEF/epNR 90/10	0.071
PEF/epNR 75/25	0.208
PEF/epNR 50/50	2.358
PBF/epNR 90/10	0.070
PBF/epNR 75/25	0.207
PBF/epNR 50/50	0.599

## Data Availability

The original contributions presented in this study are included in the article material. Further inquiries can be directed to the corresponding authors.
